# Photoreceptor deficits appear at eye opening in *Rs1* mutant mouse models of X-linked retinoschisis

**DOI:** 10.1016/j.exer.2024.109872

**Published:** 2024-03-19

**Authors:** Matthew J. Tarchick, Craig Beight, Paul B. Bonezzi, Neal S. Peachey, Jordan M. Renna

**Affiliations:** a Department of Biology, University of Akron, Akron, OH, USA; b Department of Ophthalmic Research, Cole Eye Institute, Cleveland Clinic, Cleveland, OH, 44195, USA; c Research Service, VA Northeast Ohio Healthcare System, Cleveland, OH, 44106, USA; d Department of Ophthalmology, Cleveland Clinic Lerner College of Medicine of Case Western Reserve University, Cleveland, OH, 44106, USA

**Keywords:** Electroretinography, Outer retina, X-linked retinoschisis, Retinoschisin, Retinal development

## Abstract

X-linked retinoschisis (XLRS) is an early onset degenerative retinal disease characterized by cystic lesions in the middle layers of the retina. These structural changes are accompanied by a loss of visual acuity and decreased contrast sensitivity. XLRS is caused by mutations in the gene *Rs1* which encodes the secreted protein Retinoschisin 1. Young *Rs1*-mutant mouse models develop key hallmarks of XLRS including intraretinal schisis and abnormal electroretinograms. The electroretinogram (ERG) comprises activity of multiple cellular generators, and it is not known how and when each of these is impacted in *Rs1* mutant mice. Here we use an *ex vivo* ERG system and pharmacological blockade to determine how ERG components generated by photoreceptors, ON-bipolar, and Müller glial cells are impacted in *Rs1* mutants and to determine the time course of these changes. We report that ERG abnormalities begin near eye-opening and that all ERG components are involved.

## 1. Introduction

Mutations in *Retinoschisin* (*RS1*) underlie X-linked retinoschisis (XLRS), an early onset retinal degeneration (Sauer et al., 1997). XLRS is characterized by the presence of intraretinal schisis, decreased visual acuity and contrast sensitivity, and a marked reduction in the b-wave of the flash ERG (Peachey et al., 1987; Tanino et al., 1985). Rodent *Rs1* mutants recapitulate many features of the human phenotype and provide valuable opportunities to understand basic pathophysiological features of XLRS (Eleftheriou et al., 2022; Heymann et al., 2022; Jablonski et al., 2005; Liu et al., 2019). The ERG is comprised of multiple components, including those generated by photoreceptors, ON-bipolar cells, and Müller glial cells (MGCs) which can be dissected pharmacologically (Bonezzi et al., 2020, 2023; Frishman and Steinberg, 1989). We use an *ex vivo* isolated retina preparation to evaluate the response properties of these major components in adult *Rs1* mutant mice.

In addition to examining the adult retina, we take advantage of the high signal-to-noise feature of the *ex vivo* ERG to address a second question: When is the onset of the XLRS ERG phenotype? *Rs1* mutants as young as postnatal day (P)15 have a particularly severe disease phenotype (Eleftheriou et al., 2022; Liu et al., 2019). We examine *Rs1* mutants at earlier ages. Our main findings are (a) all major cell components of the ERG-photoreceptor, ON-bipolar cell, and MGC- are affected by XLRS in mature and young animals and (b) photoreceptors are the earliest cells involved.

## 2. Methods

### Ethical approval

This study was performed in accordance with the guidelines of the Institutional Animal Care and Use Committee (IACUC) at the University of Akron.

### 2.1. Genetic mouse models of XLRS

Experiments were conducted using three mouse models of XLRS. *Rs1*^*KO*^, *Rs1*^*R141C*^, *Rs1*^*C59S*^ and a wildtype. Wildtype and mutant mice were maintained on the same background strain as described previously (Liu et al., 2019). In brief, *Rs1*^*KO*^ models were generated by a lacZ replacement of *Rs1* exons 1–3. *Rs1*^*R141C*^ and *Rs1*^*C59S*^ are point mutants for mutations that underlie human disease. Mice were maintained on a 12:12 light:dark cycle with *ad lib* access to food and water. Mice were dark-adapted for at least 6 h before experiments after which all experimental procedures were performed under dim-red LED light. Mice younger than P14 were sacrificed with 0.2 mL pentobarbital sodium (Fatal plus; Vortech Pharmaceuticals) followed by cervical dislocation. Older mice were sacrificed with CO_2_ asphyxiation followed by cervical dislocation. Eyes were removed, placed onto a cotton ball in a Petri dish containing Locke’s bubbled with 95% O_2_ and 5% CO_2_. Retinas were physically separated from the retinal pigmented epithelium (RPE) and prepared for recordings.

### 2.2. Electroretinography

Transretinal recordings were obtained using the *ex vivo* recording configuration described previously (Bonezzi et al., 2020, 2023). ERGs were recorded utilizing silver chloride pellets with positive terminal leads plugged into the bottom chamber (ganglion cell side) and reference terminals plugged into the top chamber (photoreceptor cell side). Before recordings, voltage offsets and resistances between the electrode pairs were measured to assure optimal recording conditions.

Using Clampex 10.0 (Molecular Devices), signals were amplified x1,000 (Multiclamp 700B; Molecular Devices), using a low-pass 8-pole Bessel filter at 300 Hz, digitized at 10 kHz, (Digidata 1550B; Molecular Devices) and stored.

Light stimulus protocols were controlled using Clampex 10.0 (Molecular Devices). All responses represent an average of three successive flashes. Light-responses were evoked by 1–4 ms single light pulses from a LED band-passed at 505 ± 20 nm. Photon flash intensity varied from 5 to 50,000 photons/um^2^. Light evoked responses were obtained from wildtype and mutant strains at P9, P11, P13, and P14 as well as adults older than P30.

### 2.3. Pharmacology

ERGs were recorded while retinae were perfused with three separate solutions. First by normal Locke’s, then by Locke’s containing just barium chloride BaCl_2_ (100 μM), then by Locke’s containing BaCl_2_, L-AP4 (14 μM), and aspartic acid (AA) (100 μM).

Each solution was mixed separately, then applied sequentially for the duration of the flash family experiment. After the flash family was finished, we began the perfusion of the next solution and waited 10–20 min before beginning the flash protocol.

### 2.4. Component subtraction

Each stimulus condition was collected in triplicate, and then averaged off-line. Response components were isolated by subtracting intensity matching traces from the same retina maintained in the different perfusion solutions using custom written computer software.

The MGC component was derived by subtracting responses recording in normal Locke’s from those recorded in Locke’s + BaCl_2_. MGC responses were measured from the pre-stimulus baseline to the negative trough. The ON-bipolar component was derived by subtracting the experiments collected with Locke’s + BaCl_2_ from Locke’s + BaCl_2_ +AA + L-AP4. The ON-bipolar response was measured from the pre-stimulus baseline to positive peak.

The response-properties of the photoreceptor component were derived from measurement of the Locke’s + BaCl_2_ +AA + L-AP4 condition. Photoreceptor responses were calculated from the pre-stimulus baseline to the negative trough. The a-wave of the ERG includes two phases, a negative hyperpolarization “nose” followed by a slower recovery phase (Robson and Frishman, 2014). The nose reflects extracellular currents in the outer nuclear layer (ONL) (Robson et al., 2003). The subsequent phase reflects the closure of cyclic nucleotide gated channels in the photoreceptor outer segments, the amplitude of which was measured from baseline.

#### 2.4.1. Intensity response fitting

Intensity response functions were calculated by plotting ERG component amplitude as a function of light intensity. Intensity response functions were fit to a Hill type equation:

$$\frac{R}{R_{max}}=\frac{I^{n}}{I^{n}+k}$$

Where **I** is the intensity of light, **n** is the coefficient of the slope, **K** is the intensity of light needed to elicit a half-maximal response, and **R**_**max**_ is the maximum response.

### 2.5. Custom code, plotting and statistics

All analysis was conducted using a custom written Julia package located at https://github.com/mattar13/ElectroPhysiology.jl. Detailed instructions for using the package are in the documentation. Fits were generated using LsqFit.jl. Statistics were done using HypothesisTests.jl as unequal variance t-tests. Figures were made and formatted using PyPlot.jl.

## 3. Results

### 3.1. Ex vivo ERG for pharmacological isolation of cellular components

**Fig. 1.**
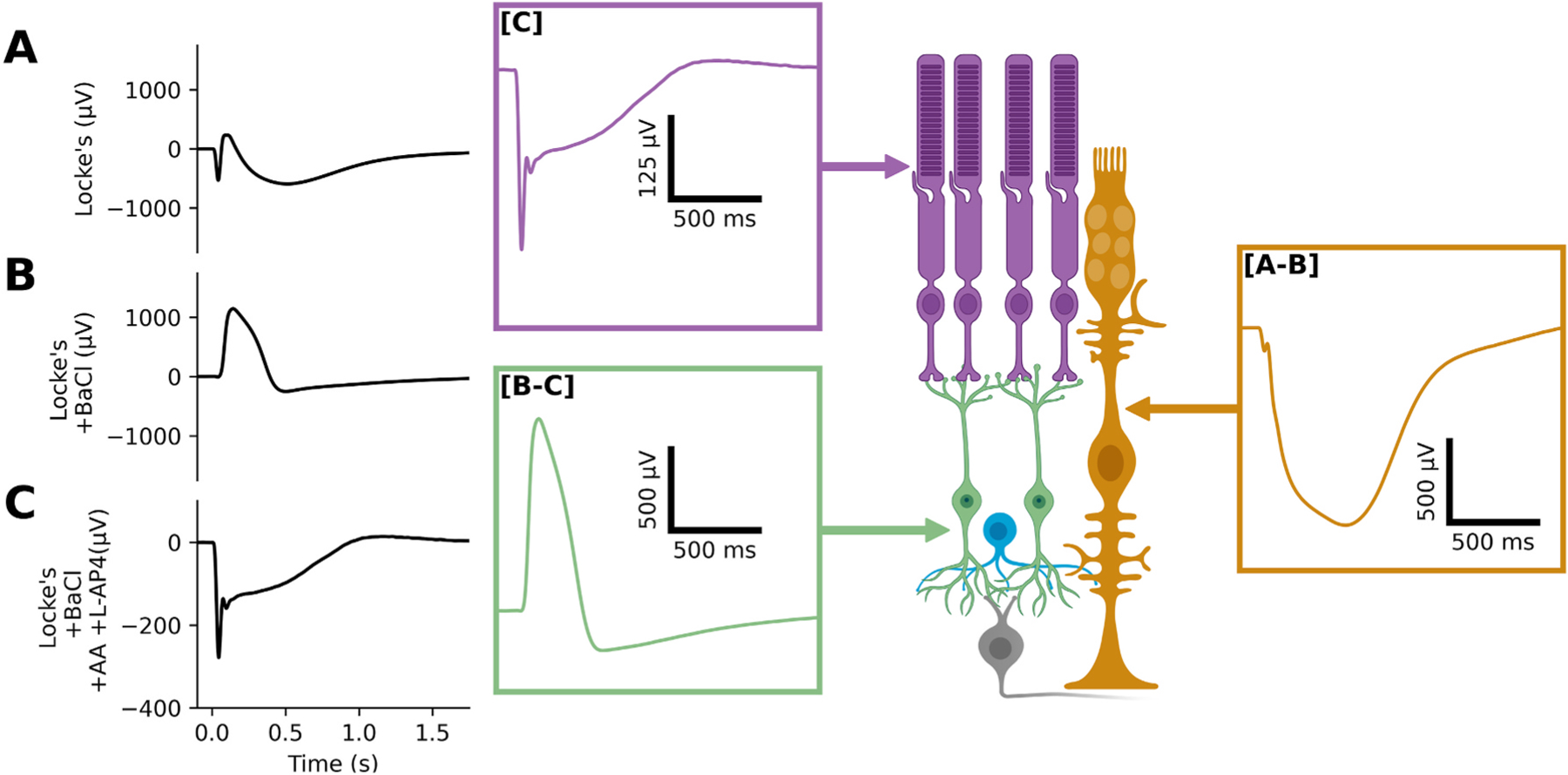
Component subtraction The sequential addition of pharmacological reagents isolates components contributing to the ERG waveform. **(A)** The baseline ERG results in a quick negative transient, followed by a positive component, followed by a slower negative component. **(B)** With the addition of barium chloride (BaCl_2_), the slow negative component attributed to the MGC is removed. **(C)** Further addition of L-AP4 and aspartic acid (AA) remove the positive component leaving only the photoreceptors. By using a subtraction method **[B-C]** results in the positive ON-bipolar, and **[A-B]** results in the slow negative MGC component. These are the main visible components of the *ex vivo* ERG. The cartoon image of retinal cells was rendered using BioRender.com.

In this study we sought to leverage the advantages of the *ex vivo* ERG to understand the impact of *Rs1* mutations on retinal function. Fig. 1 presents a representative waveform from a wildtype animal using three different pharmacological treatments. We first recorded a series of light responses from retinas perfused with Locke’s solution. The waveform shown in Fig. 1A was obtained with a 1514.6 photons/μm^2^ stimulus and includes responses from all major cell populations that contribute to the *ex vivo* ERG.

We repeated the flash series in retina perfused with Locke’s + BaCl_2_. Fig. 1B shows that the addition of BaCl_2_ to the perfusion solution impacted the waveform evoked by the 1514.6 photons/μm^2^ stimulus, such that the overall response became more positive, and the positive component had a broader timeframe. The addition of BaCl_2_ blocks the inward potassium currents of the MGC cell population (Reichelt and Pannicke, 1993).

We repeated the flash series a third time, with retinas perfused with Locke’s + BaCl_2_ + L-AP4 + AA. L-AP4 activates mGluR6 which removes the positive ON-bipolar response and AA blocks the OFF-bipolar cells responses (Slaughter and Miller, 1981; Vinberg et al., 2009). Pharmacological isolation using L-AP4 and AA removes bipolar cell responses, resulting in a negative waveform (Fig. 1C).

We next used these flash series to extract cell-specific ERG components (Bonezzi et al., 2020; Leinonen et al., 2020). Subtraction of ERGs obtained from retinas perfused with BaCl_2_ (Fig. 1B) from those perfused with Locke’s solution (Fig. 1A) yields an estimate of the MGC contribution (Fig. 1A–B). Subtraction of ERGs obtained from retinas perfused with BaCl_2_ (Fig. 1B) from those perfused with BaCl_2_ + AA + LAP4 (Fig. 1C) yields an estimate of the ON bipolar cell contribution (Fig. 1B–C). This subtraction will include an OFF-bipolar component which we do not consider further as these cells make a minor contribution to the dark-adapted mouse ERG (Akula et al., 2019).

Finally, responses obtained from retinas perfused with BaCl_2_ + AA + LAP4 (Fig. 1C) provide an estimate of the photoreceptor response. Because the RPE has been physically removed, the c-wave is not present. We then examined the response properties of these cell-specific components in wildtype and *Rs1* mutant mice.

### 3.2. Responses of all cell components are reduced in adult Rs1 mice

**Fig. 2.**
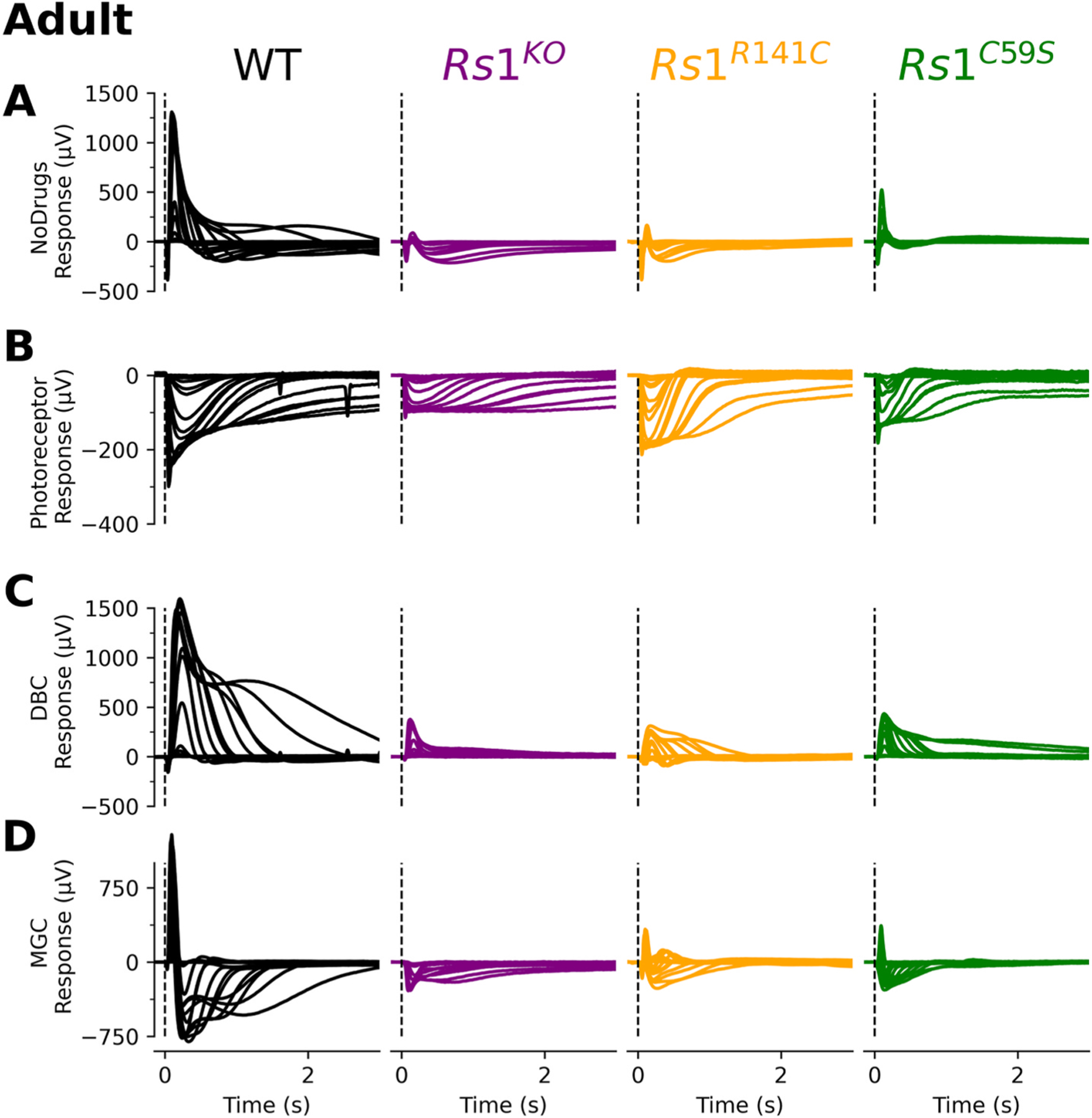
Adult Traces of XLRS mutation models. Each flash intensity family was broken down based on light intensity. A vertical dotted line in each panel indicates the location of the test flash (t = 0ms), while a horizontal dotted line represents baseline (0 mV). **(A)** Baseline responses were shown without the presence of drugs. **(B)** For photoreceptor responses, recorded retinal responses were measured in the presence of BaCl_2_, L-AP4, and aspartic acid resulting in only the light-evoked photoreceptor response. **(C)** ON-bipolar and **(D)** MGC cell responses were derived from subsequent pharmacology trials.

We recorded ERGs from adult wildtype and *Rs1* mutant mice under three perfusion conditions to examine the impact of mutant RS1 on the major components of the ERG. Fig. 2 compares a representative data set from a wildtype mouse and each of the *Rs1* mutants. For all models, the upper row (Fig. 2A) compares responses obtained when the retinas were perfused with Locke’s solution. In comparison to wildtype, the *Rs1* mutants had distinct ERG abnormalities consistent with other animal studies (Heymann et al., 2022; Jablonski et al., 2005; Liu et al., 2019). The lower rows present the isolated photoreceptor (Fig. 2B), ON-bipolar (Fig. 2C) and MGC (Fig. 2D) components. In comparison to wildtype, each isolated component obtained from *Rs1* mutant mice was reduced in amplitude.

**Fig. 3.**
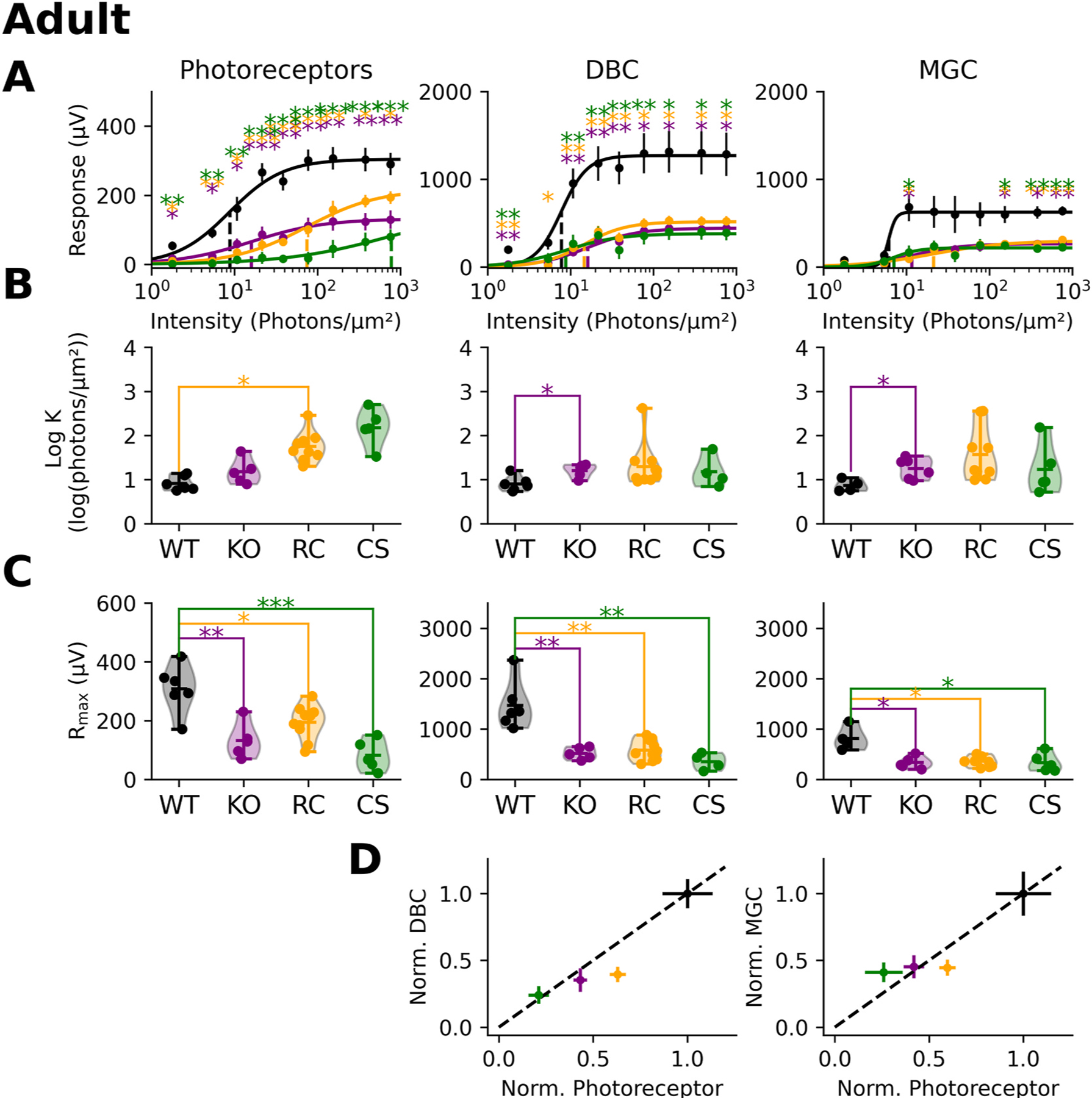
Response stats of Adult XLRS models **(A)** Response amplitudes were plotted in comparison to the light intensity and intensity-response functions were fitted. The dotted line represents the summarizing statistic, the sensitivity value for the entire collated dataset. **(B)** The sensitivity value (K) for individual experiments was calculated. The response amplitudes were summarized for each component, and statistics were calculated. **(C)** The maximal response (*R*_*max*_) amplitude was calculated for photoreceptors, ON-bipolar, and MGC components. **(D)** The R_max_ of the photoreceptors were plotted against the ON-bipolar R_max_ and MGC R_max_. Each R_max_ was normalized to the photoreceptor, ON-bipolar, or MGC wildtype response.

Fig. 3A plots intensity-response functions for the derived photoreceptor (left), ON-bipolar (middle) and MGC (right) components. Statistically significant differences from wildtype were seen across the intensity range for all models, which tended to have similar response functions (Asterisks indicate p-value levels: * = 0.01 < p < 0.05, ** = 0.001 < p < 0.01, and *** = p < 0.001). We fit the intensity response functions for each individual animal to derive the **K** and extract the **R**_**max**_ parameter. Overall, the **K** parameter was similar to the wildtype for each of the *Rs1* mutants (Fig. 3B). In comparison, the **R**_**max**_ parameter was consistently and significantly reduced for all *Rs1* mutants in comparison to wildtype (Fig. 3C). The slope parameter, **n**, did not differ between wildtype and *Rs1* mutant mice (data not shown).

Photoreceptors convey their responses to ON-bipolar cells through a specialized ribbon synapse (Thoreson, 2021). Prior studies of *Rs1* mutants have noted abnormalities in various ribbon synapse proteins (Ou et al., 2015), suggesting that this is a second site of dysfunction beyond the photoreceptors. To examine this, we plotted the values of **R**_**max**_ for the photoreceptor and ON-bipolar cell components against one another (Fig. 3D). The diagonal line indicates a proportional change from wildtype for both components. Data falling below the line would indicate that the ON-bipolar response is reduced disproportionately to the photoreceptor signal. For each *Rs1* mutant, data fall along the diagonal line indicating a proportional reduction in photoreceptor and ON-bipolar components. We examined the MGC component using the same approach (Fig. 3D right) and reached a similar conclusion.

### 3.3. Emergence of functional abnormalities in Rs1 mutant mice coincides with eye opening

*Rs1* mouse mutants display ERG deficits as early as P15 (Liu et al., 2019), and intraretinal schisis as early as P13. At P10-P11 however, inner retinal physiology appears comparable to the wildtype (Eleftheriou et al., 2022). We address the question of the onset of XLRS in the outer retina by examining the response properties of *Rs1* mutants before (P9, P11) and during eye-opening (P13, P14). Recording *in vivo* ERGs on developing animals is possible but difficult due to the small body size and corneal surface (Bakall et al., 2003; Keeler et al., 1928). Here we took advantage of the excellent signal-to-noise properties of the *ex vivo* ERG (Bonezzi et al., 2018, 2020, 2023) using the same protocol used above for the adult studies.

**Fig. 4.**
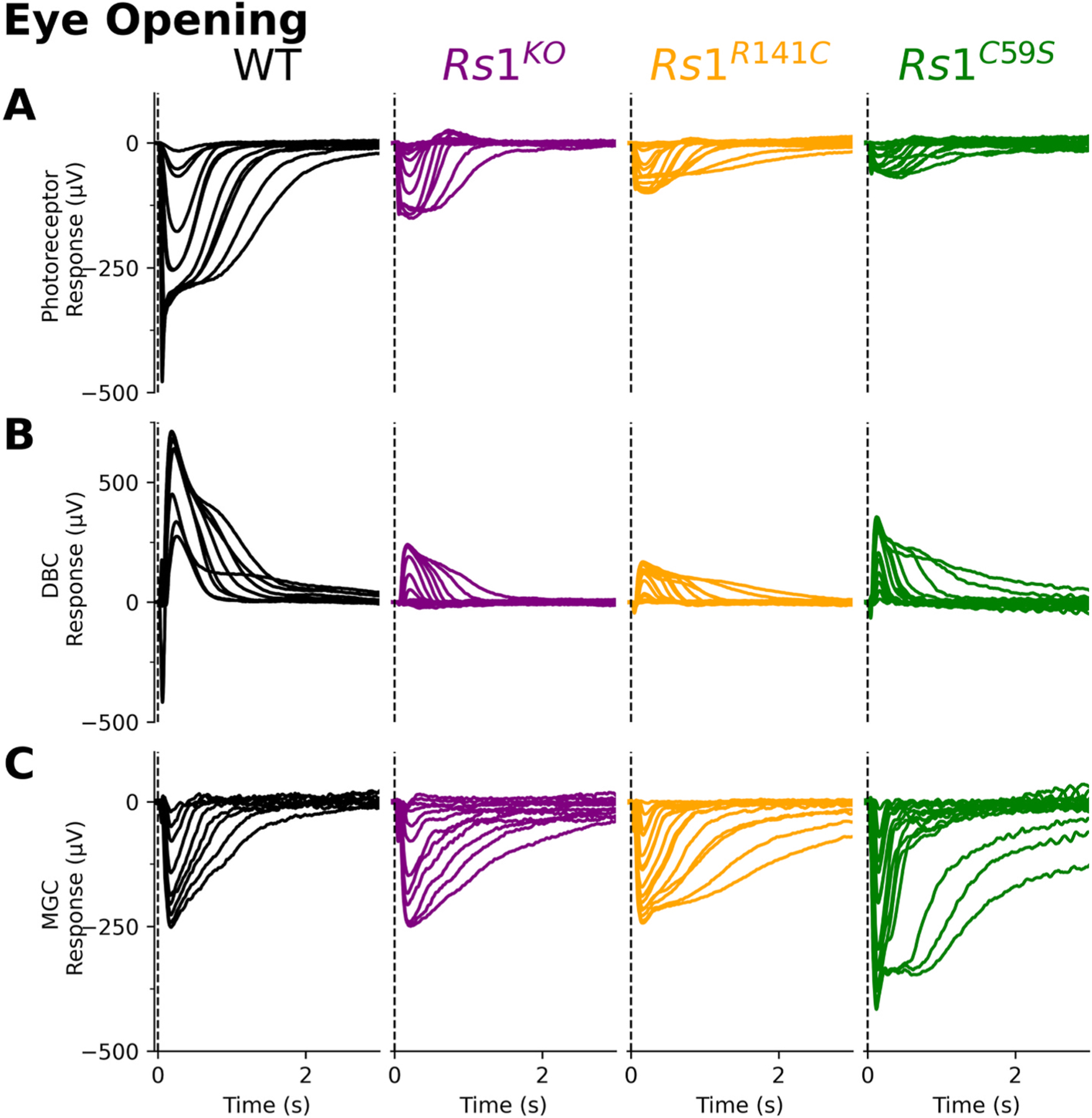
Eye-opening traces of XLRS mutation models. Each flash intensity family was broken down based on light intensity. A vertical dotted line in each panel indicates the location of the test flash (t = 0ms), while a horizontal dotted line represents baseline (0 mV). **(A)** For photoreceptor responses, recorded retinal responses were measured in the presence of BaCl_2_, L-AP4, and Aspartic acid resulting in only the light-evoked photoreceptor response. **(B)** ON-bipolar and **(C)** MGC cell responses were derived from subsequent pharmacology trials.

Fig. 4 presents the isolated ERG components obtained at eye-opening (P13–14). The photoreceptor (Fig. 4A), ON-bipolar (Fig. 4B), and MGC (Fig. 4C) components were extracted using the same subtraction method for adults. Unlike adults, deficits at eye-opening were not uniformly observed across components and mutations. Instead, each ERG component and *Rs1* mutation exhibited distinct effects.

**Fig. 5.**
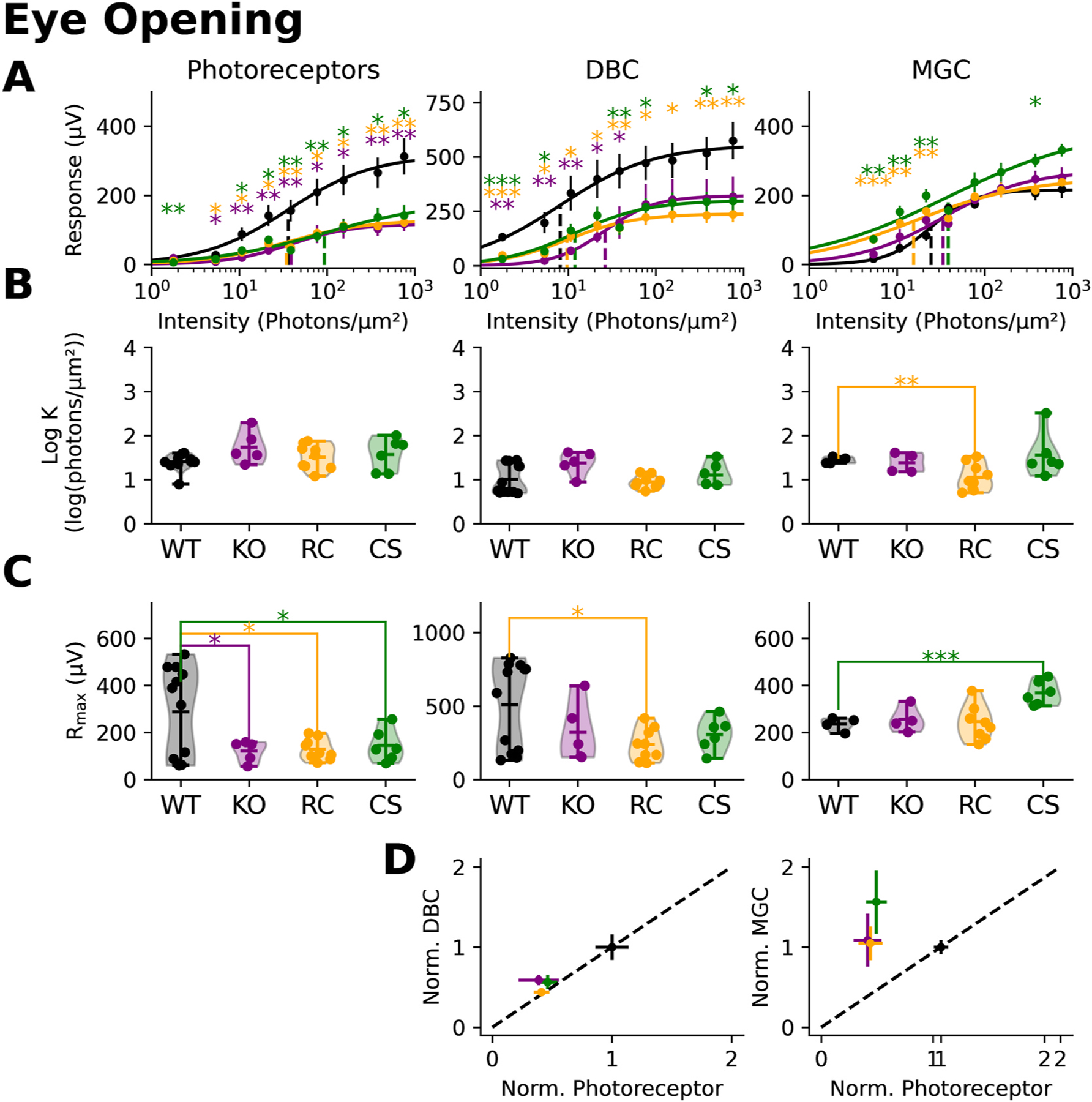
Response kinetics during eye-opening **(A)** Response amplitudes were plotted in comparison to the light intensity and intensity-response functions were fitted. The dotted line represents the summarizing statistic, the sensitivity value for the entire collated dataset. **(B)** The sensitivity value (K) for individual experiments was calculated. The response amplitudes were summarized for each component, and statistics were calculated. **(C)** The maximal response (*R*_*max*_) amplitude was calculated for photoreceptors, ON-bipolar, and MGC components. **(D)** The R_max_ of the photoreceptors were plotted against the ON-bipolar R_max_. Each R_max_ was normalized to the photoreceptor or ON-bipolar wildtype response.

Intensity response functions (Fig. 5A) were used to determine sensitivity (Log **K**; Fig. 5B) and response amplitudes (**R**_**max**_; Fig. 5C). The most prominent difference occurred in the photoreceptors, where the *Rs1* mutant response amplitudes were consistently smaller in all the Rs1 mutants (**R**_**max**_; Fig. 5C left). No changes were seen in sensitivity (Log **K**; Fig. 5B left).

ON-bipolar responses displayed more variability between mutant models (Fig. 5C). Only the *Rs1*^*R141C*^ displayed significantly reduced amplitudes. Despite this, ON-bipolar responses are nearly proportional to photoreceptor responses (Fig. 5D).

The MGC responses on the other hand, are either not affected or paradoxically enhanced at these early timepoints. While the intensity-response functions overlapped with the wildtype for *Rs1*^*KO*^ and *Rs1*^*R141C*^ models, *Rs1*^*C59S*^ seemed to have enhanced sensitivity (Log **K**; Fig. 5A and B right). **R**_**max**_ values were also significantly larger in the *Rs1*^*C59S*^ model (Fig. 5C, right). MGC responses were proportionally larger than those observed for photoreceptors in all mutant models (Fig. 5D).

**Fig. 6.**
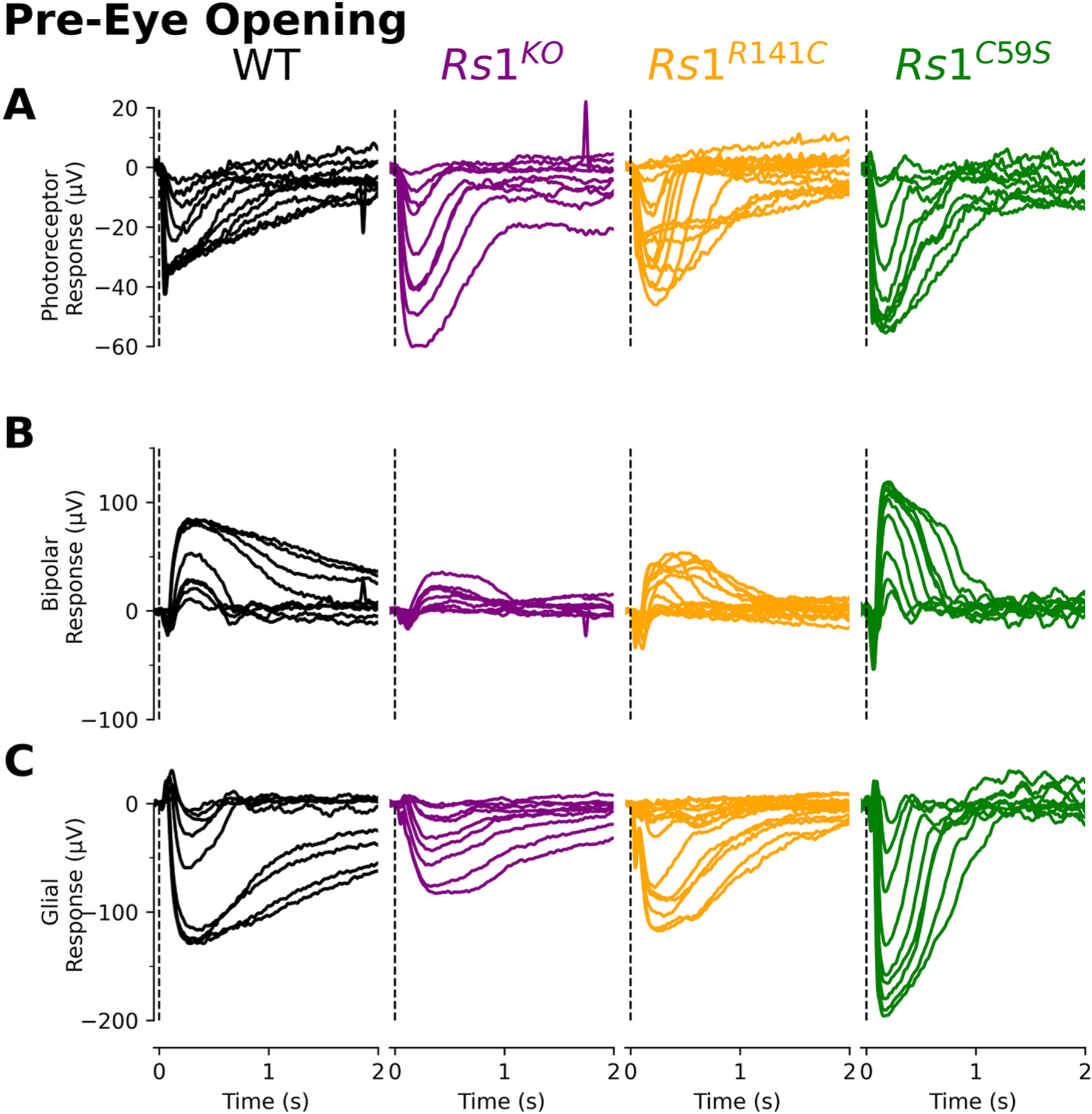
Pre-eye opening traces of XLRS mutation models. Each flash intensity family was broken down based on light intensity. A vertical dotted line in each panel indicates the location of the test flash (t = 0ms), while a horizontal dotted line represents baseline (0 mV). **(A)** For photoreceptor responses, recorded retinal responses were measured in the presence of BaCl_2_, L-AP4, and Aspartic acid resulting in only the light-evoked photoreceptor response. **(B)** ON-bipolar and **(C)** MGC cell responses were derived from subsequent pharmacology trials.

**Fig. 7.**
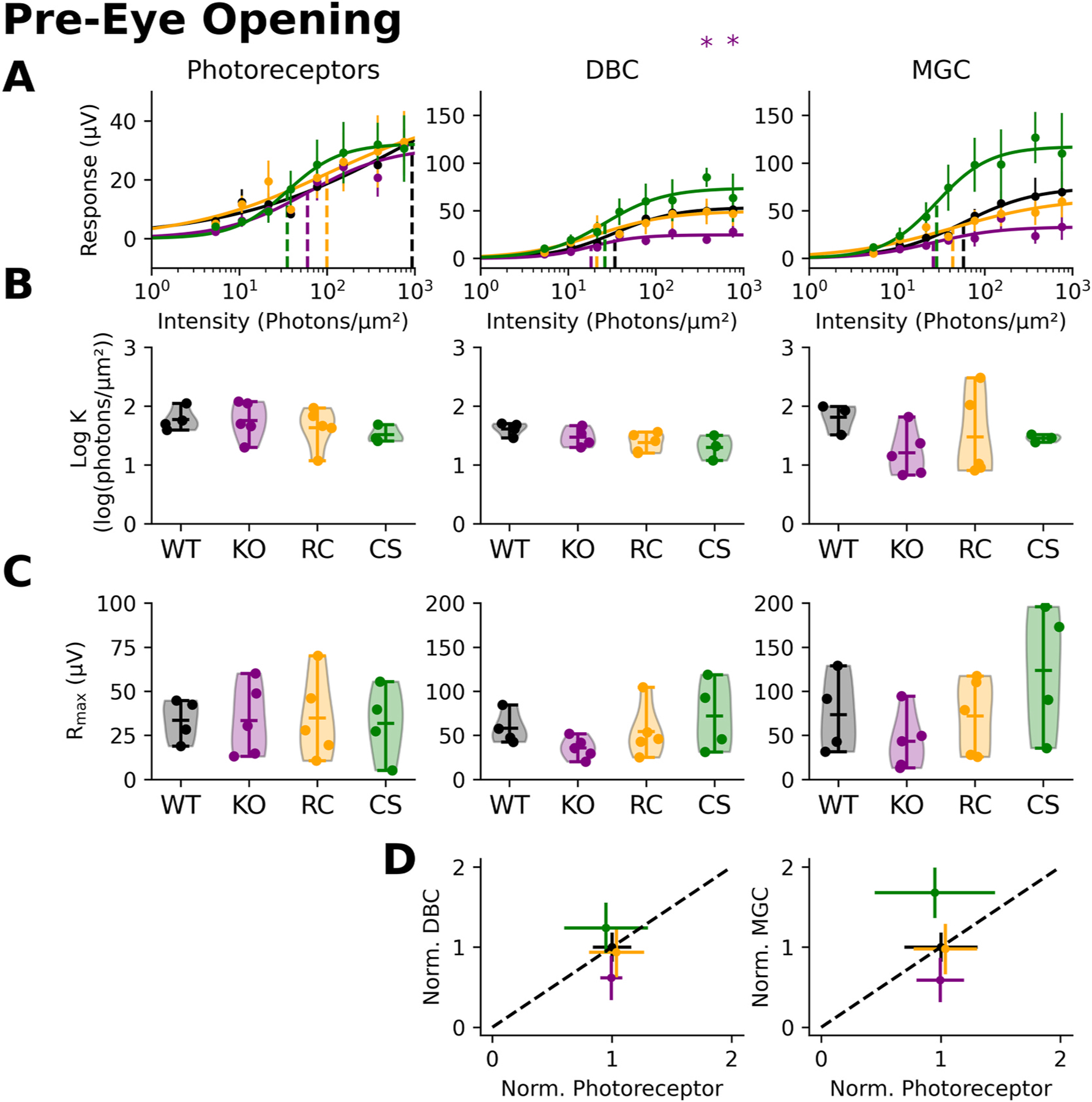
Response kinetics before eye opening. **(A)** Response amplitudes were plotted in comparison to the light intensity and intensity-response functions were fitted. The dotted line represents the summarizing statistic, the sensitivity value for the entire collated dataset. **(B)** The sensitivity value (K) for individual experiments was calculated. The response amplitudes were summarized for each component, and statistics were calculated. **(C)** The maximal response (*R*_*max*_) amplitude was calculated for photoreceptors, ON-bipolar, and MGC components. **(D)** The R_max_ of the photoreceptors were plotted against the ON-bipolar R_max_. Each R_max_ was normalized to the photoreceptor or ON-bipolar wildtype response.

Fig. 6 presents representative waveforms of components obtained before eye opening (P9-P11). The kinetics of the RS1 mutation models (*Rs1*^*KO*^, *Rs1*^*R141C*^*, Rs1*^*C59S*^) are similar at developmental timepoints. The photoreceptor (Fig. 6A), ON-bipolar (Fig. 6B), and MGC (Fig. 6C) responses have the same polarity as the adult. However, there are no clear differences between the responses. There are also no differences in any of the intensity-response functions (Fig. 7A), sensitivity (Log **K**; Fig. 7B) or the (**R**_**max**_; Fig. 7C). The amplitude proportions are all reasonably close as well (Fig. 7D).

### 3.4. Comparison of XLRS mutations over time

**Fig. 8.**
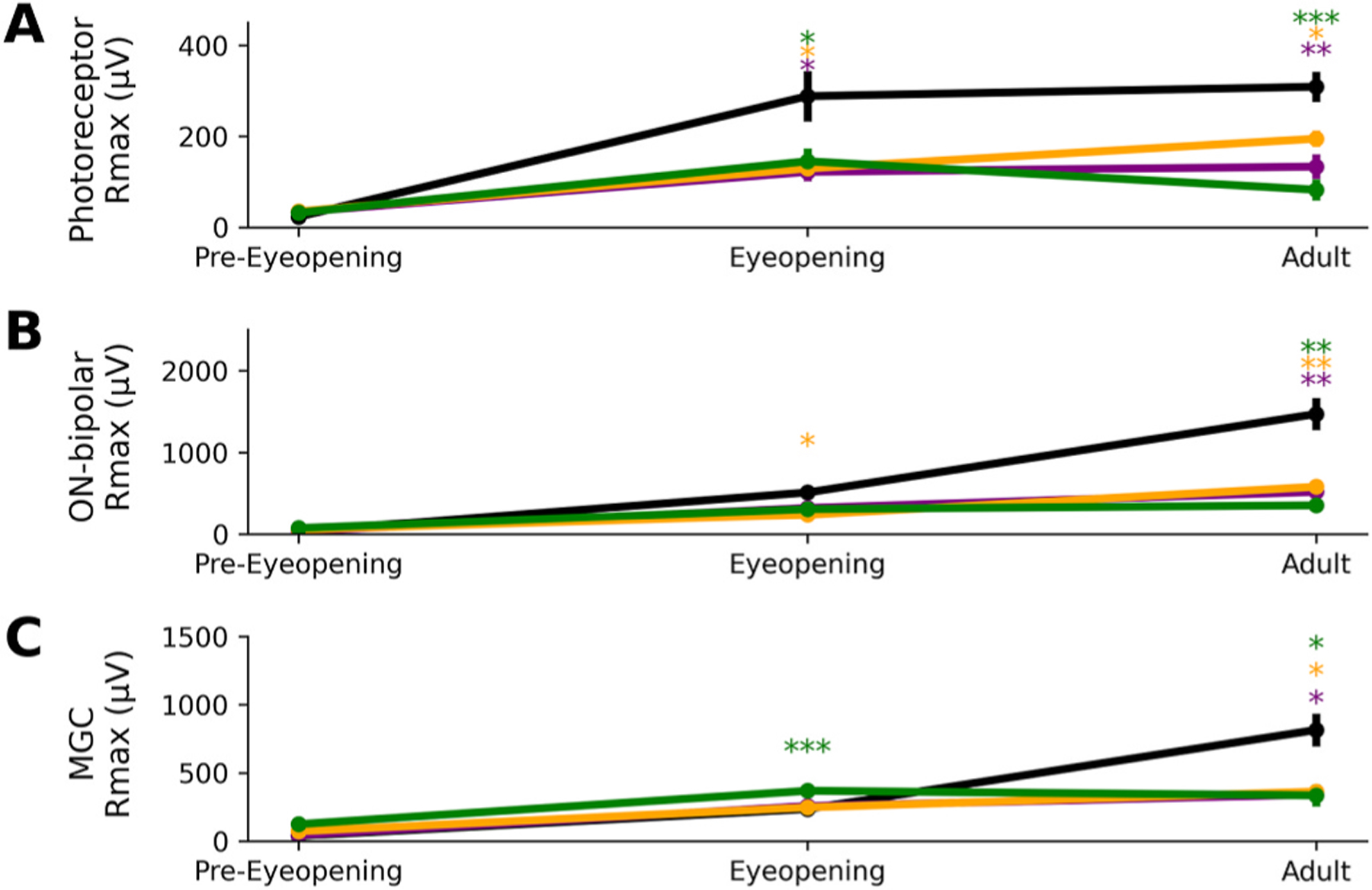
Development of the XLRS phenotype over time. The progression of the XLRS phenotype was monitored over time. The maximum response was averaged with anywhere from 5 to 18 retinae. Each developmental timepoint has plot insets to represent a zoomed in perspective. Individual components were compared such as **(A)** photoreceptor responses, **(B)** subtracted ON-bipolar cell responses.

To determine the earliest effect of the XLRS mutation, component specific **R**_**max**_ values for each genotype were compared before eye-opening (P11), during eye-opening (P13-P14) and the adult animal (Fig. 8). Wildtype data is consistent with our previous findings: the photoreceptor response increases from P11 to eye-opening (Fig. 8A) and then stabilizes (Bonezzi et al., 2018, 2023). Out of the three mutations, the *Rs1*^*KO*^ maintains the same stability, despite having a smaller amplitude. The *Rs1*^*C59S*^ decreases in amplitude, and the *Rs1*^*R141C*^ amplitude gets larger, but never quite reaching the amplitude of the wildtype.

In wildtype animals, the ON-bipolar component (Fig. 8B) and MGC component (Fig. 8C) amplitudes continue to increase after eye-opening. The *Rs1* mutants lack this increase in amplitude. The adult ON-bipolar cell deficits mirror those of photoreceptors deficits. *Rs1*^*C59S*^ decreases in amplitude after eye-opening, and *Rs1*^*R141C*^ increases. These findings indicate that photoreceptors are the first cell population to have reduced function because of mutant *Rs1*, and that ON-bipolar cells mirror those deficits.

## 4. Discussion

In this study, we used the *ex vivo* ERG to examine response properties of isolated cell populations affected by *Rs1* mutations across development. The main findings are: **(A)** The ON-bipolar response abnormalities of *Rs1* mutant mice mirror those of photoreceptors, suggesting that ON-bipolar cells carry forward the abnormal photoreceptor response without any additional insult. **(B)** The light-evoked MGC response of *Rs1* mutant mice is also reduced in parallel with the photoreceptor response in adults, but is larger than seen in wildtype animals at early disease stage; **(C)** the response properties of the *Rs1* mutant retina are relatively normal before eye-opening after which abnormalities appear beginning in the photoreceptors. These results are relevant to understanding the role of RS1 in the retina and during retinal development.

### 4.1. Advantages of the ex vivo electroretinogram

Previous publications have established an improved technique for the *ex vivo* ERG (Bonezzi et al., 2020; Kolesnikov and Kefalov, 2012; Vinberg et al., 2009). Because of the early onset of XLRS and multiple functional cell types involved (Eleftheriou et al., 2022; Liu et al., 2019; Molday et al., 2001; Takada et al., 2004), the *ex vivo* ERG provides a powerful method to study the cell populations affected by XLRS. By utilizing cell-specific blockers and providing a stable recording environment, the isolated preparation allows for pharmacological isolation of individual components. The high signal-to-noise allows for the detection of smaller developmental responses (Bonezzi et al., 2020). We previously used this technique to track the development of rod photoreceptors (Bonezzi et al., 2018), cone photoreceptors, and ON-bipolar cells (Bonezzi et al., 2023) before eye-opening.

### 4.2. XLRS begins in the photoreceptors but affects other components

Mutant Rs1 affects the function of many of the cells that contribute to the mouse ERG waveform. Adult Rs1 mutant response amplitudes are smaller for the photoreceptor, ON-bipolar cell, and MCG components (Figs. 2A and 3C) indicating reduced function in these cell populations. Ultrastructural analysis localizes Rs1 in the inner segments of the photoreceptors (Molday et al., 2007). Rs1 also has been found to co-precipitate with L-type voltage gated calcium channels (L-VGCC) which are important to photoreceptor function (Shi et al., 2017). Expression patterns of several other gene markers are decreased in XLRS including *Rho* (Rhodopsin), *Gnat1* (Rod Transducin), *Cnga* (cyclic nucleotide gated channel alpha subunit), and *Arr* (Arrestin) (Ambrosio et al., 2023). Many of these proteins play a direct role in phototransduction indicating that photoreceptor defects may be the initiation point of the disease’s phenotype.

### 4.3. Early effects of XLRS are present at eye opening and are exacerbated thereafter

Eye opening (P12–14) is a crucial timepoint for retinal development and maturation. Many other physiological events coincide with eye opening. For example, spontaneous retinal waves are being replaced with light responses (Blankenship and Feller, 2010), and the bipolar response reaches its half maximal threshold (Bonezzi et al., 2023). During this time, there is a sharp increase in mGluR6 between P8 and P14 and that trend continues until P20 (Anastassov et al., 2019). It is likely that much of the synaptic development done after this point in the wildtype animal involves strengthening and maintaining already present connections.

*Rs1* is first expressed in the ganglion cell layer as early as P1 and protein expression is visible at P14 (Liu et al., 2019). Ocular coherence tomography (OCT) data after eye-opening (P15) indicates that schisis is seen, and there is an accompanied decreased in the ERG b-wave (Liu et al., 2019). We found abnormalities in photoreceptor function at P13–14 (Figs. 4A and 3C). At this timepoint, there was a proportional decrease in both photoreceptors and ON-bipolar cells indicating that the signaling is altered in a top-down method (Fig. 3D).

The ON-bipolar deficits caused by XLRS propagate into inner retinal deficits. In mice with XLRS, spontaneous retinal ganglion cell activity is present after eye-opening (Eleftheriou et al., 2022). This also occurs in mice lacking vesicular glutamate release (Blankenship et al., 2009), and mice with no ON-bipolar activity *Nxy*^*Nob*^ (Demas et al., 2006). In XLRS, because of the weakness of the photoreceptor to ON-bipolar circuit response, the ON-bipolar may not provide the inner retina with the necessary glutamate source to terminate retinal waves completely. It is likely that the normal strengthening of downstream connections that occurs during eye-opening may not be able to properly occur resulting in low signal-to-noise, and low visual acuity.

In young *Rs1* mutants the MGC response was larger than in wildtype animals. To our knowledge few papers have characterized the MGC responses using the *ex vivo* ERG (Leinonen et al., 2020), and none have characterized them throughout development. At earlier timepoints, MGC function seems relatively spared (Fig. 8C). Because the ERG is a summation of these three components, *in vivo* results (which cannot isolate the slow negative component) may appear smaller. Altered Müller glial cell responses in early-stage XLRS may be linked to disruptions in the non-synaptic interactions mediated by retinoschisin (Reid et al., 2003). The enhanced or unaffected Müller glial cell responses observed in early-stage XLRS models may reflect a compensatory mechanism or disruption in the active transcytosis of retinoschisin (Reid and Farber, 2005).

While it is likely they follow the development of the ON-bipolar cells, future studies may provide more insight. MGC responses appear to be initially spared from *Rs1* mutations, with amplitudes like or even surpassing wildtype animals.

### 4.4. Comparison of ex vivo and prior in vivo data

In addition to the presence of intraretinal schisis, a hallmark signature associated with XLRS mutations is a ‘negative’ ERG (Heymann et al., 2022), indicative of a greater reduction of the b-wave as compared to the a-wave. This ERG phenotype is replicated *in vivo* in the mouse models that we studied (Liu et al., 2019). Based on this, we anticipated the isolated ON-bipolar component would be reduced to a greater extent than the isolated photoreceptor component. In contrast to this prediction, we found the *ex vivo* ERG components were reduced to similar extents, both in adults (Fig. 3D) and during development (Figs. 5D and 7D). An important difference between the *in vivo* and *ex vivo* systems is that the *ex vivo* retinae were superfused. Thus, a portion of the b-wave reduction seen *in vivo* could reflect an extracellular factor impacting ON-bipolar cell function. Extracellular factors contributing to *in vivo* ERG deficits have been described in other mouse models. For example, previous studies of *MCT3* mutant mice reported a decreased ERG a-wave (Daniele et al., 2008). Yet when retinal tissue was isolated and perfused, photoreceptor responses were comparable in mutant and control mice (Daniele et al., 2008). Daniele et al. proposed that abnormal lactate transport resulted in a pH change altering the photoreceptor response *in vivo*, but not impacting the photoresponse under conditions of experimental perfusion. In future work it would be important to determine whether some factor(s) present in the intact retina might contribute to the *Rs1* mutant b-wave abnormalities observed *in vivo*.

## 5. Implications

XLRS is an attractive model for gene therapies, primarily due to its distinct genetic cause, the *Rs1* gene. The specificity of the mutation in XLRS lends itself to targeted gene therapy approaches, such as viral vector-mediated RS1 gene delivery and genome editing techniques like CRISPR-Cas9. Gene therapies for XLRS have had benefits on ERG phenotypes. One therapeutic approach has been to use of the adeno-associated virus type 8-RS1 (AAV8-RS1) vector (Ou et al., 2015). Delivering the vector to the outer retina to *Rs1*^*KO*^ mouse retina resulted in a selective improvement in ERG b-wave amplitude. For review of present methods see (Vijayasarathy et al., 2022).

Our findings emphasize two critical aspects. First, the photoreceptor emerges as the primary site of dysfunction, central to the pathogenesis of XLRS. Therapeutics specifically targeting photoreceptor resilience and functionality might be pivotal in addressing the root cause of the disorder. Secondly, our findings underscore the significance of the developmental timeline of XLRS. Photoreceptor functional deficits begin to manifest around the time of eye opening, highlighting a crucial timepoint for therapeutic intervention. This early phase in the disease’s progression represents a period where targeted gene therapy could be most impactful, potentially preventing or significantly mitigating the progression of XLRS.

## Data Availability

Data will be made available on request.

## Abbreviations

AA: Aspartic acid
AAV8-RS1: Adeno-associated virus type 8-RS1
BaCl_2_: Barium chloride
ERG: Electroretinogram
L-AP4: L-2-amino-4-phosphonobutyric acid
L-VGCC: L-type voltage gated calcium channels
MGC: Müller glial cells
OCT: Ocular coherence tomography
RS1: Retinoschisin
XLRS: X-linked retinoschisis

## References

[R1] Akula JD, Ambrosio L, Howard FI, Hansen RM, Fulton AB, 2019. Extracting the ON and OFF contributions to the full-field photopic flash electroretinogram using summed growth curves. Exp. Eye Res 189 10.1016/j.exer.2019.107827. PMC6956400 31600486

[R2] Ambrosio L, Akula JD, Harman JC, Arellano IA, Fulton AB, 2023. Do the retinal abnormalities in x-linked juvenile retinoschisis include impaired phototransduction? Exp. Eye Res 234 10.1016/j.exer.2023.109591. 37481224

[R3] Anastassov IA, Wang W, Dunn FA, 2019. Synaptogenesis and synaptic protein localization in the postnatal development of rod bipolar cell dendrites in mouse retina. J. Comp. Neurol 527, 52–66. 10.1002/cne.24251. 28547795 PMC5745277

[R4] Bakall B, Marmorstein LY, Hoppe G, Peachey NS, Wadelius C, Marmorstein AD, 2003. Expression and localization of bestrophin during normal mouse development. Invest. Ophthalmol. Vis. Sci 44, 3622–3628. 10.1167/iovs.03-0030. 12882816

[R5] Blankenship AG, Feller MB, 2010. Mechanisms underlying spontaneous patterned activity in developing neural circuits. Nat. Rev. Neurosci 11, 18–29. 10.1038/nrn2759. 19953103 PMC2902252

[R6] Blankenship AG, Ford KJ, Johnson J, Seal RP, Edwards RH, Copenhagen DR, Feller MB, 2009. Synaptic and extrasynaptic factors governing glutamatergic retinal waves. Neuron 62, 230–241. 10.1016/j.neuron.2009.03.015. 19409268 PMC2807181

[R7] Bonezzi PJ, Stabio ME, Renna JM, 2018. The development of mid-wavelength photoresponsivity in the mouse retina. Curr. Eye Res 43, 666–673. 10.1080/02713683.2018.1433859. 29447486 PMC6094161

[R8] Bonezzi PJ, Tarchick MJ, Moore BD, Renna JM, 2023. Light drives the developmental progression of outer retinal function. J. Gen. Physiol 155 10.1085/jgp.202213262. PMC10336150 37432412

[R9] Bonezzi PJ, Tarchick MJ, Renna JM, 2020. Ex vivo electroretinograms made easy: performing ergs using 3d printed components. J. Physiol 598, 4821–4842. 10.1113/JP280014. 32886799 PMC9009099

[R10] Daniele LL, Sauer B, Gallagher SM, Pugh EN, Philp NJ, 2008. Altered visual function in monocarboxylate transporter 3 (Slc16a8) knockout mice. Am. J. Physiol. Cell Physiol 295, C451–C457. 10.1152/ajpcell.00124.2008. 18524945 PMC2518420

[R11] Demas J, Sagdullaev BT, Green E, Jaubert-Miazza L, McCall MA, Gregg RG, Wong ROL, Guido W, 2006. Failure to maintain eye-specific segregation in nob, a mutant with abnormally patterned retinal activity. Neuron 50, 247–259. 10.1016/j.neuron.2006.03.033. 16630836

[R12] Eleftheriou CG, Corona C, Khattak S, Alam NM, Ivanova E, Bianchimano P, Liu Y, Sun D, Singh R, Batoki JC, Prusky GT, McAnany JJ, Peachey NS, Romano C, Sagdullaev BT, 2022. Retinoschisin deficiency induces persistent aberrant waves of activity affecting neuroglial signaling in the retina. J. Neurosci 42, 6983–7000. 10.1523/JNEUROSCI.2128-21.2022. 35906066 PMC9464019

[R13] Frishman LJ, Steinberg RH, 1989. Intraretinal analysis of the threshold dark-adapted ERG of cat retina. J. Neurophysiol 61, 1221–1232. 10.1152/jn.1989.61.6.1221. 2746322

[R14] Heymann JB, Vijayasarathy C, Fariss RN, Sieving PA, 2022. Advances in understanding the molecular structure of retinoschisin while questions remain of biological function. Prog. Retin. Eye Res 10.1016/j.preteyeres.2022.101147. PMC10185713 36402656

[R15] Jablonski MM, Dalke C, Wang X, Lu L, Manly KF, Pretsch W, Favor J, Pardue MT, Rinchik EM, Williams RW, Goldowitz D, Graw J, 2005. An ENU-induced mutation in Rs1h causes disruption of retinal structure and function. Mol. Vis 11, 569–581. 16088326

[R16] Keeler CE, Sutcliffe E, Chaffee EL, 1928. A description of the ontogenetic development of retinal action currents in the house mouse. Proc. Natl. Acad. Sci. U.S. A 14, 811–815. 10.1073/pnas.14.10.811. 16587413 PMC1085718

[R17] Kolesnikov AV, Kefalov VJ, 2012. Transretinal erg recordings from mouse retina: rod and cone photoresponses. JoVE 10.3791/3424. PMC3460592 22453300

[R18] Leinonen H, Pham NC, Boyd T, Santoso J, Palczewski K, Vinberg F, 2020. Homeostatic plasticity in the retina is associated with maintenance of night vision during retinal degenerative disease. Elife 9, 1–27. 10.7554/eLife.59422. PMC7529457 32960171

[R19] Liu Y, Kinoshita J, Ivanova E, Sun D, Li H, Liao T, Cao J, Bell BA, Wang JM, Tang Y, Brydges S, Peachey NS, Sagdullaev BT, Romano C, 2019. Mouse models of x-linked juvenile retinoschisis have an early onset phenotype, the severity of which varies with genotype. Hum. Mol. Genet. 28, 3072–3090. 10.1093/hmg/ddz122. 31174210 PMC6737296

[R20] Molday LL, Hicks D, Sauer CG, Weber BH, Molday RS, 2001. Expression of x-linked retinoschisis protein rs1 in photoreceptor and bipolar cells. Invest. Ophthalmol. Vis. Sci 42, 816–825. 11222545

[R21] Molday LL, Wu WWH, Molday RS, 2007. Retinoschisin (rs1), the protein encoded by the x-linked retinoschisis gene, is anchored to the surface of retinal photoreceptor and bipolar cells through its interactions with a na/k atpase-sarm1 complex. J. Biol. Chem 282, 32792–32801. 10.1074/jbc.M706321200. 17804407

[R22] Ou J, Vijayasarathy C, Ziccardi L, Chen S, Zeng Y, Marangoni D, Pope JG, Bush RA, Wu Z, Li W, Sieving PA, 2015. Synaptic pathology and therapeutic repair in adult retinoschisis mouse by aav-rs1 transfer. J. Clin. Invest 125, 2891–2903. 10.1172/JCI81380. 26098217 PMC4563692

[R23] Peachey NS, Fishman GA, Derlacki DJ, Brigell MG, 1987. Psychophysical and electroretinographic findings in x-linked juvenile retinoschisis. Arch. Ophthalmol 105, 513–516. 10.1001/archopht.1987.01060040083038. 3566604

[R24] Reichelt W, Pannicke T, 1993. Voltage-dependent K+ currents in Guinea pig Müller (glial) cells show different sensitivities to blockade by Ba2+. Neurosci. Lett 155, 15–18. 10.1016/0304-3940(93)90663-6. 8361658

[R25] Reid SNM, Farber DB, 2005. Glial transcytosis of a photoreceptor-secreted signaling protein, retinoschisin. Glia 49, 397–406. 10.1002/glia.20131. 15538749

[R26] Reid SNM, Yamashita C, Farber DB, 2003. Retinoschisin, a photoreceptor-secreted protein, and its interaction with bipolar and Müller cells. J. Neurosci 23, 6030–6040. 10.1523/JNEUROSCI.23-14-06030.2003. 12853421 PMC6740352

[R27] Robson JG, Frishman LJ, 2014. The rod-driven a-wave of the dark-adapted mammalian electroretinogram. Prog. Retin. Eye Res 39, 1–22. 10.1016/j.preteyeres.2013.12.003. 24355774 PMC3939025

[R28] Robson JG, Saszik SM, Ahmed J, Frishman LJ, 2003. Rod and cone contributions to the *a* -wave of the electroretinogram of the macaque. J. Physiol 547, 509–530. 10.1113/jphysiol.2002.030304. 12562933 PMC2342654

[R29] Sauer CG, Gehrig A, Warneke-Wittstock R, Marquardt A, Ewing CC, Gibson A, Lorenz B, Jurklies B, Weber BH, 1997 Oct. Positional cloning of the gene associated with X-linked juvenile retinoschisis. Nature Genetics 17 (2). 10.1038/ng1097-164, 164–70. 9326935

[R30] Shi L, Ko ML, Ko GYP, 2017. Retinoschisin facilitates the function of l-type voltage-gated calcium channels. Front. Cell. Neurosci 11 10.3389/fncel.2017.00232. PMC5550728 28848397

[R31] Slaughter MM, Miller RF, 1981. 2-amino-4-phosphonobutyric acid: a new pharmacological tool for retina research. Science 211, 182–185. 10.1126/science.6255566. 6255566

[R32] Takada Y, Fariss RN, Tanikawa A, Zeng Y, Carper D, Bush R, Sieving PA, 2004. A retinal neuronal development wave of retinoschisin expression begins in ganglion cells during layer formation. Invest. Ophthalmol. Vis. Sci 45, 3302–3312. 10.1167/iovs.04-0156. 15326155

[R33] Tanino T, Katsumi O, Hirose T, 1985. Electrophysiological similarities between two eyes with x-linked recessive retinoschisis. Doc. Ophthalmol 60, 149–161. 10.1007/BF00158030. 4042821

[R34] Thoreson WB, 2021. Transmission at rod and cone ribbon synapses in the retina. Eur. J. Physiol 473, 1469–1491. 10.1007/s00424-021-02548-9. PMC8680117 33779813

[R35] Vijayasarathy C, Sardar Pasha SPB, Sieving PA, 2022. Of men and mice: human X-linked retinoschisis and fidelity in mouse modeling. Prog. Retin. Eye Res 87, 100999 10.1016/j.preteyeres.2021.100999. 34390869

[R36] Vinberg FJ, Strandman S, Koskelainen A, 2009. Origin of the fast negative erg component from isolated aspartate-treated mouse retina. J. Vis 9 10.1167/9.12.9, 9–9. 20053100

